# The effect of inulin as a fat substitute on the physicochemical and sensory properties of chicken sausages

**DOI:** 10.1002/fsn3.585

**Published:** 2018-01-22

**Authors:** Fereshteh Alaei, Mohammad Hojjatoleslamy, Seyyed Majid Hashemi Dehkordi

**Affiliations:** ^1^ Department of Food Science and Technology Islamic Azad University Shahrekord Branch Shahrekord Iran

**Keywords:** fat substitution, inulin, physicochemical properties, sausages, sensory properties

## Abstract

Due to its high thermal resistance and compatibility with the sausage emulsion system, the long‐chain inulin can be used as a fat substitute in the formulation of this product. This study was conducted to investigate the effect of inulin on the physicochemical, textural, and sensory properties of chicken sausages. The study included treatments of 25%, 50%, 75%, and 100% substitution. After preparing the samples, their physicochemical, textural, calorimetric, and sensory properties were evaluated. The treatment of 100% substitution of inulin had the maximum amount of sugar (29.90%), moisture (72.63%), protein (51.34), ash (6.95%), and salt (4.02%) (dry basis). The fat content was decreased with the increased levels of inulin substitution (p < .05). The increased amount of inulin reduced hardness, cohesiveness, gumminess, and stringiness, but increased springiness and chewiness up to the 25% substitution of inulin. The highest color difference and hue angle were related to 100% substitution treatment. The sensory evaluation of the samples showed that with the increase in the amount of inulin, the mean scores of the factors including color, appearance, and texture were increased, but the mean scores of smell and mouthfeel were decreased. Overall, the substitution of the entire fat existing in the formulation of the sausage with inulin led to the best physicochemical, textural, colorimetric, and sensory results. The use of inulin could be recommended as a fat substitute in the formulation of chicken sausages.

## 1 INTRODUCTION

Nowadays, the importance of meat and its products is not hidden from anyone. Due to its iron, zinc, protein, sodium, magnesium, potassium, calcium, essential amino acids, such as histidine, tryptophan, phenylalanine, threonine, valine, leucine, isoleucine, and lysine contents, and its desirable taste, red meat is consumed by millions of people every day. On the other hand, with the increasing development of human knowledge, the consumption of meat and meat products has become more common than before.

Sausages are among meat products, which are popular; they are usually produced by red meat and chicken. The amount of fat in meat products affects the sensory properties of the product, playing a major role in the creation of a creamy state, desirable appearance, deliciousness, texture acceptability, and a feeling of satiety. Hence, formulating a low‐fat meat product without any change in its taste, mouthfeel, and other organoleptic characteristics is a very detailed and specialized process (Maghsudi, 2002). Sausages produced and marketed in Iran contain at least 40% meat (Anonymous 2000; Naseri, 1980). The meat products produced in Iran fall in the group of emulsion sausages (Naseri, 1980), which are usually classified based on their formulation, process temperature, type of coverage, and diameter (Savic, 1985).

Studies have shown that there is a direct relationship between the type of diet and the risk of some diseases such as cancer, obesity, and cardiovascular diseases. Hence, the growing concerns about the potential risks associated with the consumption of high‐fat foods have caused the food industry to begin to develop new formulations and modify traditional food products into those with lower fat contents (Alenson‐Carbonel, Fernandez‐Lopez, Perez‐Alvarez, & Kuri, 2005). Nowadays, fat substitutes have provided new solutions to the production of a variety of new low‐fat foods; these products have a pleasant taste and texture like high‐fat products, but do not contain unnecessary calories and cholesterol.

Inulin is a soluble plant fiber, which contains types of oligosaccharides and monosaccharides. Due to the formation of a gel during mixing with water, inulin can easily be used as a fat substitute in food products. The gel obtained from inulin has a creamy and appropriate consistency, creating a mouthfeel of fat in low‐fat food products. Results of studies have shown that inulin is not only an appropriate substitute for fat in food products, but also produces a very low calorie, about 1–1.5 kcal per gram (Ahmed, Miller, Lyon, Vaughters, & Reagan, 1990; Coussement & Frank, 2001).

In the literature review conducted, no information was found regarding the use of this additive as a fat substitute in the formulation of sausages and its effect on the qualitative characteristics of this product. Accordingly, the present study was conducted with the aim of evaluating the physicochemical, textural, color, and sensory properties of sausage samples produced through the substitution of fat with inulin.

## 2 MATERIALS AND METHODS

### 2.1 The preparation of the samples

The sausage sample with 40% chicken meat contained zero percent inulin, and its production was in accordance with the requirements specified in the Iranian National Standard in Shaghayegh Factory of Saman; this sample was considered as the control sample. The inulin used in this study was a kind of long‐chain inulin (Sensus, Netherlands). The chicken sausage samples were produced according to a previously proposed method (Naseri, 1980). Inulin solution was a substituent of additional vegetable oil added in 15% (w/w) in cutter, and inulin solution was prepared by adding 200 gr inulin in 7800 gr water (2.5% w/w). The amount of fat substitution was calculated by direct oil replacement through the described inulin solution.

### 2.2 Evaluating the chemical properties of the sausages

The AOAC standard method No. 991/30 was used to measure the fat content of the samples in a petroleum ether solvent (AOAC 1994), and the AOAC standard method No. 981/10 was used to measure the protein content of the samples according to the Kjeldahl method (AOAC 1983). The AOAC standard method No. 950/46 was used to measure the moisture content of the samples by using an oven at 125°C (AOAC 1991), and the AOAC standard method No. 920/153 was used to measure the ash content of the samples by employing an electrical furnace at 550°C (AOAC 1996). ISO 2917 (1999) was used to measure the pH of the samples, and a digital pH meter was used for this purpose; also, the amount of salt present in the samples was measured according to Codex Standard No. 192 (2011).

### 2.3 Evaluating the physical properties of the sausages

The factors L*, a* and b*, which correspond to the lightness, redness and yellowness of the color of the samples, respectively, were measured using a Hunter laboratory device (Ahmed et al., 1990; Candogan & Kolsarici, 2003). The following equations were used to calculate the values of ΔE (Equation (1)) and Hue angle (Equation (2)).

(1) $$\Delta E=\sqrt{{\left(L-\mathrm{Lc}\right)}^{2}+{\left(b-bc\right)}^{2}+{\left(a-\mathrm{ac}\right)}^{2}}$$

(2) $$\text{Hue angle}=\text{Arc tan}\frac{b}{a}$$

### 2.4 The texture profile analysis of the sausage samples

To investigate the textural properties of the sausages, cylindrical samples of the sausages with the dimensions of 1 × 1 × 1 were cut and put under a pressure test by CT3 Brookfield engineering, USA, using a texture profile analysis probe (TA 4/1000, 38 mm diameter) under a load of 4.5 kg. The force needed to compress the samples down to 70% of their initial height was measured under a constant speed of 200 mm/min (Vural, 2003). The hardness, springiness, cohesiveness, adhesiveness, and chewiness of the samples were evaluated in this study. Among these factors, hardness, cohesiveness, springiness, and stringiness are related to the nature of food, but gumminess and chewiness are relevant to these factors, as expressed in Equations (3) and (4) (Steffe, 1992).

(3) $$\text{Gumminess}\;=\;{\text{Hardness}}^{*}\text{Cohesiveness}$$

(4) $$\begin{array}{cc} \text{Chewiness} & \;=\;{\text{Gumminess}}^{*}\text{Springiness}\\ & ={\text{Hardness}}^{*}{\text{Cohesiveness}}^{*}\text{Springiness} \end{array}$$

### 2.5 Sensory tests

The sensory evaluation was carried out on the produced samples by 10 trained panelists. The characteristics tested included smell, taste, color, view, appearance, texture (softness and hardness), color, mouthfeel, and overall acceptability. The test conditions were quite identical for the sensory panelists; also, to enhance the accuracy of the sense of taste, water and bread were used between each two samples being tested. The test was designed in a 9‐point hedonic scale.

### 2.6 Statistical analysis of data

All the tests were carried out in three replications; in order to analyze the data, SPSS Software, version 20, was used. The design was a complete randomized block one, and the means comparison was carried out using one‐way ANOVA and Duncan's multiple range test (p < .05).

## 3 RESULTS

Table 1 displays the mean physicochemical properties of the sausage samples in different treatments. According to the obtained results, the increased levels of substituting fat with inulin enhanced the moisture, protein, sugar, ash, and salt content of the samples and reduced their fat content.

**Table 1:** Mean percentages of moisture, fat, protein, sugar, ash, and salt in different sausage treatments

Treatment	Biochemical characteristics (g of dry matter)					
	Moisture	Fat (dry basis)	Protein (dry basis)	Sugar (dry basis)	Ash (dry basis)	Salt (dry basis)
100%	72.63 ± 0.33^a^	0.15 ± 0^e^	51.34 ± 0.16^a^	29.90 ± 0.15^b^	6.95 ± 0.01^a^	4.02 ± 0.25^a^
75%	69.13 ± 0.33^b^	2.66 ± 0.33^d^	46.37 ± 0.15^b^	25.62 ± 0.026^c^	5.72 ± 0.1^b^	3.39 ± 0.24^b^
50%	64.33 ± 0.33^c^	7.50 ± 0^c^	40.65 ± 0.036^c^	21.49 ± 0.1^d^	5.51 ± 0.09^c^	3.24 ± 0.24^c^
25%	61.30 ± 0^d^	11.20 ± 0^b^	37.64 ± 0.086^d^	20.41 ± 0^e^	5.17 ± 0^d^	3.10 ± 0.26^d^
Control	58.03 ± 0.33^e^	15.16 ± 0.33^a^	32.24 ± 0.07^e^	32.01 ± 0.08^a^	4.05 ± 0.13e	2.43 ± 0.13^e^

Dissimilar letters in each column are indicative of a significant difference at an error probability level of 5%.

Tables 2, 3, 4 indicate the textural properties of the sausage samples during 30 days of storage. The results showed that the increased level of inulin reduced the textural hardness of the samples. Nevertheless, the highest level of hardness was related to the control sample (3,216.3 g), and the hardness of the sausage samples was moderately increased during the storage period. According to the results, the cohesiveness degree did not change with the increased level of inulin on the day zero. Changes in the cohesiveness degree were also irregular on different days of storage, and the increased level of inulin reduced gumminess in the sausage treatments; moreover, the level of gumminess was increased during the storage period. The results also showed that springiness was increased with the increased level of substitution up to 25% inulin, and then decreased. During the storage period, it was also increased until the day 20, but then it was decreased. The increased level of inulin substitution reduced the stringiness of the sausage samples, and the level of stringiness was increased during 30 days of storage. The chewiness of the sausage samples was decreased with the increased level of substituting fat with inulin. Changes in the chewiness of the samples were not regular during the storage period.

**Table 2:** Evaluating the hardness and cohesiveness of the sausage samples in different treatments on different days of storage

Treatment	Values of textural hardness during the storage period (g)				Values of cohesiveness during the storage period			
	Day 0	Day 10	Day 20	Day 30	Day 0	Day 10	Day 20	Day 30
100%	1709.1 ± 0.29^Dc^	2593.8 ± 0.40^Cb^	2881.8 ± 0.058^Ab^	2689.5 ± 0.60^Bb^	0.835 ± 0.004^Aa^	0.770 ± 0.011^Ab^	0.790 ± 0.011^Aa^	0.780 ± 0.0^Aa^
75%	1836.5 ± 0.62^Cc^	1994.2 ± 0.62^Bc^	2078.1 ± 1.19^Ac^	1949.5 ± 0.75^Bc^	0.840 ± 0.011^Aa^	0.790 ± 0.017^Ba^	0.760 ± 0.017^Cb^	0.790 ± 0.014^Ba^
50%	2463.7 ± 2.55^Db^	2954.5 ± 5.32^Bb^	2812 ± 28.86^Cb^	3198.8 ± 3.84^Aa^	0.840 ± 0.0^Aa^	0.800 ± 0.011^Ba^	0.750 ± 0.011^Cb^	0.810 ± 0.017^Ba^
25%	3213.8 ± 3.47^Ba^	3285.3 ± 1.12^Ba^	3319.6 ± 2.62^Ab^	3050.1 ± 25.45^Ca^	0.840 ± 0.79^Aa^	0.800 ± 0.72^Ba^	0.810 ± 0.81^Ba^	0.820 ± 0.77^Ba^
Control	3216.3 ± 30.79^Ca^	3402.1 ± 3.21^Ba^	3534.6 ± 1.41^Aa^	3140.6 ± 1.38 ^Da^	0.830 ± 0.005^Aa^	0.790 ± 0.011^Aa^	0.760 ± 0.016^Bb^	0.750 ± 0.007^Bb^

Dissimilar capital letters in each row are indicative of a significant difference at an error probability level of 5%. Dissimilar small letters in each column are indicative of a significant difference at an error probability level of 5%.

**Table 3:** Evaluating the gumminess and springiness of the sausage samples in different treatments on different days of storage

Treatment	Values of textural gumminess during the storage period (g)				Values of springiness during the storage period (mm)			
	Day 0	Day 10	Day 20	Day 30	Day 0	Day 10	Day 20	Day 30
100%	1427.09 ± 0.032^Bc^	1997.22 ± 0.023^Ac^	2276.62 ± 0.088^Ab^	2097.81 ± 2.29^Aa^	0.25 ± 0.020^Bb^	0.34 ± 0.005^Ba^	1.27 ± 0.017^Ab^	0.45 ± 0.014^Bd^
75%	1542.66 ± 1.038^Ac^	1575.41 ± 1.91^Ad^	1579.35 ± 0.96^Ac^	1540.10 ± 13.24^Ab^	0.195 ± 0.003^Bb^	0.21 ± 0.023^Bb^	0.87 ± 0.011^Ac^	1.10 ± 0.075^Ac^
50%	2069.50 ± 0.34^Ab^	2363.60 ± 4.87^Ba^	2109 ± 5.88^Cb^	2591.02 ± 7.46 ^Da^	2.15 ± 0.014^Aa^	0.37 ± 0.011^Ba^	0.60 ± 0.028^Bc^	0.05 ± 0.009^Ce^
25%	2603.17 ± 0.79^Aa^	2628.24 ± 0.25.23^Ba^	2688.87 ± 0.27.61^Ca^	2501.08 ± 0.23.12 ^Da^	0.25 ± 0.017^Bb^	0.18 ± 0.017^Bb^	0.45 ± 0.017^Bc^	2.01 ± 0.040^Ab^
Control	2669.52 ± 16.33^Aa^	2687.65 ± 1.37^Ba^	2686.29 ± 29.17^Ba^	2355.45 ± 3^Bb^	0.20 ± 0.59^Bb^	0.18 ± 0.023^Bb^	3.16 ± 0.017^Aa^	2.84 ± 0.028^Aa^

Dissimilar capital letters in each row are indicative of a significant difference at an error probability level of 5%. Dissimilar small letters in each column are indicative of a significant difference at an error probability level of 5%.

**Table 4:** Evaluating the stringiness and chewiness of the sausage samples in different treatments on different days of storage

Treatment	Values of textural stringiness during the storage period (mm)				Values of chewiness during the storage period (mJ)			
	Day 0	Day 10	Day 20	Day 30	Day 0	Day 10	Day 20	Day 30
100%	7.36 ± 0.017^Cb^	8.81 ± 0.011^Bb^	9.11 ± 0.023^Aa^	9.14 ± 0.017^Aa^	10503.4 ± 200.53^Cd^	17595.3 ± 408.21^Bc^	20739.8 ± 5412^Ab^	19174 ± 104.75^Ac^
75%	7.36 ± 0.017^Bb^	8.54 ± 0.034^Ab^	8.80 ± 0.072^Ab^	8.69 ± 0.041^Ab^	11353.5 ± 0.72^Bd^	13453.9 ± 3.19^Ad^	13897.8 ± 12.92^Ac^	13383.4 ± 5.13^Ad^
50%	7.49 ± 0.023^Bb^	9.20 ± 0.028^Aa^	9.03 ± 0.037^Aa^	9.31 ± 0.023^Aa^	15500.5 ± 6.14^Cc^	21745.1 ± 12.76^Bb^	19044.2 ± 12.70^Bb^	24122.3 ± 54.31^Aa^
25%	9.20 ± 0.011^Aa^	9.35 ± 0.017^Aa^	9.18 ± 0.023^Aa^	9.14 ± 0.017^Aa^	23948.5 ± 142.50^Aa^	24573.6 ± 42.61^Aa^	24683.1 ± 52.72^Aa^	22859.1 ± 38.29^Ab^
Control	7.35 ± 0.017^Bb^	7.81 ± 0.003^Bc^	8.96 ± 0.054^Aa^	8.53 ± 0.014^Ab^	19620.8 ± 6.37^Bb^	20990.1 ± 43.46^Bb^	24068.3 ± 28.57^Aa^	20091.5 ± 22.19^Bc^

Dissimilar capital letters in each row are indicative of a significant difference at an error probability level of 5%. Dissimilar small letters in each column are indicative of a significant difference at an error probability level of 5%.

**Figure 1:**
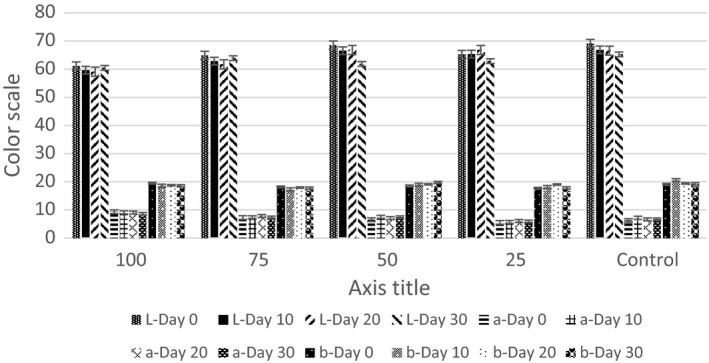
Mean changes in color parameters L*, a*, and b* during storage

Figure 1 shows the mean values for the colorimetric factors in different sausage treatments during 30 days of storage. According to the obtained results, the increased level of inulin enhanced the textural lightness of the samples up to the 50% substitution, and then reduced it; also, the textural lightness of the samples was moderately decreased during the storage period. The textural redness of the sausage samples was enhanced with the increased level of inulin substitution, and changes in the textural redness were not regular during the storage period of the samples. The increased level of substituting fat with inulin approximately increased the textural yellowness of the samples, and changes in the yellowness of the samples were not regular during the storage period.

**Figure 2:**
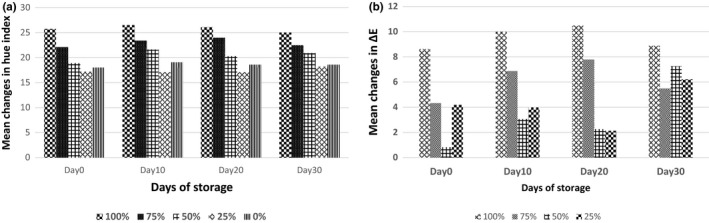
Mean changes in ΔE (a) and Hue index (b) in sausage treatments in different days of storage

Figure 2 shows ΔE changes for different sausage treatments during the storage period. The ΔE value of the samples was increased perceptibly with the increased level of inulin in a way that the treatment of 100% substitution of inulin had the highest value of ΔE in all treatments. Nevertheless, changes in the ΔE value of the samples were not regular during 30 days of storage. Figure 2 shows changes in the values of Hue index in different sausage treatments during 30 days of storage. In the present study, the values of Hue index obtained for the treatments were between 17 and 27. As expressed in Figure 2, more fat substitution caused the higher hue angle, leading to color change from red to orange, while control sample tended to be red.

Table 5 shows the mean sensory scores given to different sausage treatments. The panelists gave the highest sensory scores, on average, to the treatment of 100% substitution of fat with inulin. In other words, the increased level of inulin enhanced the scores given to the factors: color, appearance, and texture, but the mean scores given to smell were decreased.

**Table 5:** Results of the sensory evaluation for each sausage treatment

Treatment	Sensory scores				
	Mouthfeel	Texture	Appearance	Color	Smell
100%	7 ± 0.37^a^	7 ± 0.31^a^	6.2 ± 0.2^a^	7.4 ± 0.4^a^	5.6 ± 0.24^d^
75%	6.6 ± 0.24^b^	6.6 ± 0.34	6 ± 0.54^a^	7 ± 0.31^b^	5.6 ± 0.24^d^
50%	6.4 ± 0.2^b^	6.2 ± 0.2^c^	6 ± 0.31^a^	6.6 ± 0.24^c^	6 ± 0.31^c^
25%	6.4 ± 0.2^b^	6 ± 0.31^c^	5.8 ± 0.37^a^	6 ± 0.31^d^	6.4 ± 0.24^b^
Control	7 ± 0.31^a^	6 ± 0.31^c^	6.2 ± 0.2^a^	6.2 ± 0.37^d^	6.8 ± 0.37^a^

Dissimilar letters in each column are indicative of a significant difference at an error probability level of 5%.

## 4 DISCUSSION

The present study is one of the most comprehensive research project conducted on the effect of substituting fat with inulin to address its effects on the physicochemical and sensory properties of chicken sausages in different treatments. The results of this study showed that the use of inulin as a fat substitute could improve physicochemical, textural, color, and sensory properties of chicken sausages. With the enhanced level of inulin substitution, the moisture content of the sausage samples was increased (p < .05). The enhanced levels of inulin and water instead of fat, as well as using inulin and water in conjunction with each other in the formulation, were the main reasons for this increased moisture content. The presence of hydrophilic groups and the hygroscopic nature of inulin were other reasons for the increased level of moisture content in the samples. A study conducted by Huang, Tsai, and Chen (2011) showed that with the increased level of inulin, the moisture content of the sausage samples was decreased (Huang et al., 2011), which was different from the results obtained in our study. This was probably due to the different formulation of the sausage samples, as well as the type of inulin and the method of using it in the formulation. Reducing the amount of fat and substituting it with inulin were the main reasons for the decreased level of fat in the final product, which reduced the energy intake resulting from sausage consumption, thereby indicating that this was a dietary product. Menegas, Colombo Pimentel, Garcia, and Helena Prodencio (2013) showed that the addition of inulin reduced the fat content (42.4% ± 2.2%), as compared to that in the control group (45.4% ± 0.7%) and sausages with 50% fat (28.2% ± 1.7%) (Menegas et al., 2013). This result was quite similar to those obtained our study. Mendez Zamora et al. (2015) showed that the addition of 5.84% inulin to the formulation of Frankfurter improved the sensory and textural properties, and reduced the fat content of dietary sausages, which was consistent with the results obtained in our study. In addition, a study by Huang et al. (2011) showed that increasing the level of inulin from 3.5% to 7% considerably reduced the fat content in the sausage samples. Inulin caused the formation of gels through absorbing moisture, and increased the viscosity and sensory properties of the product through reducing its fat content, which improved the texture of the product. The increase in viscosity at high levels of inulin could be attributed to its high moisture absorbability and moisture retention.

The increased level of substituting fat with inulin also enhanced the protein content in the sausage samples, which was probably due to its calculation in dry matter. Moreover, in the study conducted by Menegas et al. (2013), the increased level of inulin enhanced the protein content of the sausage samples. Addition of 2.83% and 5.84% inulin to the Frankfurter samples, which was performed in the study by Mendez Zamora et al. (2015), increased the protein content in the samples; as the level of inulin was increased, the protein content was enhanced too (Mendez Zamora et al., 2015). In this sense, it was consistent with our findings. In a study conducted by Cegielka and Tambor (2012), in order to evaluate the effect of inulin in the samples of chicken burger, the results showed that the samples produced using 1%, 2%, and 3% inulin had higher protein contents than the control sample. Among these, the sample containing 3% inulin had the highest protein content, which was consistent with our findings (Cegielka & Tambor, 2012). A study conducted by Mendoza, Garcia, Casas, and Selgas (2001) also showed that the protein contents in the control sample of sausages, and treatments of 7.5%, 12%, 12.5%, and 14% inulin were 24%, 33.5%, 34.1%, 27.3%, and 27.6%, respectively. This indicated an increase in protein content up to a concentration of 12% inulin, and then a decrease in its content at higher concentrations of inulin (Mendoza et al., 2001).

The increased levels of sugar contents in the samples subjected inulin substitution were due to the carbohydrate nature of inulin. Of course, the increased level of inulin in the samples had no significant effect on increasing the sugar content (but it was expected to have). The main reason for this finding was, firstly, the calculation of sugar content in dry matter; secondly, in the sugar measurement method, the starch contained in the product was first hydrolyzed; then, the glucose content was measured using the Fehling's reagent. In addition, inulin had only one unit of glucose, causing the increased level of inulin to have no significant effect on raising the glucose content of the samples. The increased level of inulin substitution enhanced the ash contents in the studied samples. The main reason for this finding was the calculation of ash content in dry matter. A study conducted by Sojica et al. (2011) showed that the increased level of inulin in the sausage samples enhanced their ash content. The results of their study showed that samples containing inulin had higher ash contents than those without inulin (Sojica et al., 2011). In a study conducted by Mendoza et al. (2001), the results showed that by reducing the fat content in sausage samples and adding inulin, the ash content of the samples was increased. This increase was determined to be due to the calculation of ash content in dry matter. Huang et al. (2011) have also reported similar results.

The increased level of inulin reduced the textural hardness of the sausage samples. The main reason for this finding was probably the decreased levels of fat contents due to the increased levels of inulin in treatments with high percentages of inulin. Distribution of emulsifiers was the cause of changes in the textural hardness of the sausage samples during the storage period. On the first day of production, water and inulin were mixed together, but inulin gradually absorbed water and formed lattices. Then, it was given time to absorb the maximum water, forming stronger lattices which increased hardness during the storage period. Decreased levels of fat and lack of pressure‐resistant lattices in the sausage samples could be the main reasons for the decrease in the force required to compress the sausage samples; in other words, the higher softness of the sausage samples was the reason. In the study conducted by Mendoza et al. (2001) on the samples of dry fermented sausages, the results showed that the increased levels of inulin decreased the textural hardness of the samples of the produced sausages (Mendoza et al., 2001), which was consistent with our results. Similar results were reported by Huang et al. (2011), Cegielka and Tambor (2012), and Mendez Zamora et al. (2015).

The spread ability and the increase in the length of a sample before its textural failure are known as textural cohesiveness. Fats have high elasticity, resulting in the formation of strong lattices to preserve substances present in the formulation of sausages. The removal of fats from the formulation caused loss of elasticity and cohesiveness in the samples. Nevertheless, part of this cohesiveness was compensated by the substituted inulin to some extent. The main reason for the occurrence of these results could be attributed to the creation of an HLB system in which proteins practically played the role of emulsifiers. In the sausage treatments, protein contents were constant, but fat contents were decreased, which reduced the hydrogel section. Hence, at the beginning, it was expected that the three‐dimensional structure (fat, water, and emulsifier) would become disrupted, but over time, with the formation of an inulin lattice, this structure was restored in a different way. In studies carried out by Mendoza et al. (2001), Huang et al. (2011), Mendez Zamora et al. (2015), and Menegas et al. (2013), it was found that the increased levels of inulin reduced the textural cohesiveness of different types of sausage samples, which was consistent with some of our results.

The increased levels of inulin decreased the textural gumminess of the sausage samples, which was due to fat removal (considering its gumminess) and addition of inulin to the samples. Mendez Zamora et al. (2015) considered the reason to be the decreased levels of fat contents, and changes in water‐holding capacity. Considering the fact gumminess is the product of hardness and cohesiveness (Equation (3)), it can be attributed to changes in these factors. As can be seen in Table 1, hardness of the samples was decreased by the higher level of inulin content, while cohesiveness had less changes; also changes in Gumminess factor could be expressed by hardness changes and the ability of inulin to bind the ingredients of the sausage (Mendez Zamora et al., 2015). The high level of fiber present in the inulin added to the sausage was another reason for the decrease in the textural gumminess and the increase in the textural softness of sausage samples. On the other hand, the increase in the protein content allowed for interactions between proteins and polysaccharides, leading to the formation of a gel state and softness in the product (Xiong, Noel, & Moody, 1999). This gel state reduced the hardness and gumminess of the sample. Despite the increase in the moisture content, which caused an increase in the textural stringiness in the sausage samples (with the increased level of inulin), the main reason for the decreased level of stringiness in the samples is unknown. In other words, the ability of the sausage samples to return to their original shape after removing the deformation force was decreased in some of the treated samples, as compared to that in the control sample; this could be attributed to the less fat content as plasticizer in sausage. Lack of a suitable lattice, as well as removing a large amount of fat, could be the main reason for this situation. During the storage period of the sausage samples, the amount of force needed to chew and pulp the sample to swallow was enhanced due to the increase in the hardness of the samples during the storage period. The loosened textures of the samples treated with inulin reduced the amount of force required to chew and pulp the sample to be swallowed. The increased levels of inulin in some treatments made it easy to chew the sausage samples. However, this easiness was not enough to pulp the samples. Similar textural results were reported by Mendoza et al. (2001), Huang et al. (2011), and Menegas et al. (2013).

The absence of fat in the sample could be the main reason for the lighter color of the product. With the level of inulin was increased, Factor L was decreased; in other words, the color of the product went toward darkness, but it was not enough to cause the darkness of the product. The increased levels of inulin in all the treatments enhanced the textural redness of the samples. Similar results were reported by Sojica et al. (2011), Huang et al. (2011), and Mendez Zamora et al. (2015). It seems, therefore, that reducing large amounts of fat can decrease the lightness of the sausage samples. However, the addition of white inulin could compensate for the lost lightness. In general, the redder color of treatments containing higher inulin contents and lower fat contents was due to the red color of the meat used in these products. In other words, in the absence of fat, red meat increases the level of textural redness. Nevertheless, in the present study, chicken was used, and the main reason for the increase in the textural redness of the samples is unknown. The main reasons for the above findings could be the decreased level of fat and binding of a large part of the fiber present in inulin to the formulation. The increased level of inulin caused a regular increase in Hue index. This factor was also increased during the storage period; of course, there were some irregularities in this regard. In the present study, the desired angles for the inulin treatments were obtained up to 30 degrees, which represented the light pinkish red color; the reason is unknown. Jimenez‐Colmenero, Herrero, Pintado, Solas, and Ruiz Capillas (2010) showed that changes in the amounts of meat and fat, and addition of food fibers were the main reasons for the differences in color factors in the sausage samples.

With the increased level of inulin, the mean scores given by the panelists to the factors including color, appearance, and texture were increased, but the mean scores given to the smell were lower. It seemed that removal of fat, which caused good smell and mouthfeel to the consumer, was the main reason for the decline in the level of smell and mouthfeel in treatments with high levels of inulin. The results of the sensory evaluation for the texture of the sausage samples were quite similar to those obtained from the texture profile analysis tests. Both tests were indicative of the lower values of textural hardness in treatments with higher inulin contents. Similar results were reported in studies conducted by Mendez Zamora et al. (2015), Cegielka and Tambor (2012), Huang et al. (2011), and Sojica et al. (2011). They expressed that inulin caused the highest improvement in the texture and color of low‐fat sausages. In addition, each of the sensory properties, such as appearance (with no statistically significant difference), color (with a statistically significant difference), and texture (with a statistically significant difference), was enhanced at the higher concentrations of inulin.

## 5 CONCLUSION

The present study was conducted with the aim of evaluating the production of dietary sausages with a reduction in the fat content and substitution of inulin. Overall, the findings obtained from the physicochemical and sensory tests showed that, as well as the desirable effect of fat reduction, the addition of inulin to the formulation of the sausage samples, in the form of a 100% substitution, led to the best results, as compared to those of other treatments. Therefore, this formulation could be used by factories producing dietary sausages.

## ACKNOWLEDGMENTS

The authors of the present study would like to extend their sincerest thanks and appreciation to all hard‐working staff of Saman Meat Products Factory, especially the engineer Ms. Aminian; also, the staff of the Food Research Centre of Islamic Azad University, Shahrekord Branch, especially Eng. Manuchehr Momeni, should be appreciated. The study was sponsored by Islamic Azad University, Shahrekord Branch.

## CONFLICT OF INTEREST

The authors declare that there is no conflict of interest regarding the publication of this paper.
